# Classification of Patients’ Judgments of Their Physicians in Web-Based Written Reviews Using Natural Language Processing: Algorithm Development and Validation

**DOI:** 10.2196/50236

**Published:** 2024-08-01

**Authors:** Farrah Madanay, Karissa Tu, Ada Campagna, J Kelly Davis, Steven S Doerstling, Felicia Chen, Peter A Ubel

**Affiliations:** 1 Sanford School of Public Policy Duke University Durham, NC United States; 2 Center for Bioethics and Social Sciences in Medicine University of Michigan Medical School Ann Arbor, MI United States; 3 Fuqua School of Business Duke University Durham, NC United States; 4 School of Medicine University of Washington Seattle, WA United States; 5 Center for Advanced Hindsight Duke University Durham, NC United States; 6 Department of Sociology University of California, Los Angeles Los Angeles, CA United States; 7 Department of Population Health Sciences Duke University School of Medicine Durham, NC United States; 8 Department of Medicine Stanford University Stanford, CA United States; 9 GrantScout San Francisco, CA United States

**Keywords:** web-based physician reviews, patient judgments, RoBERTa, natural language processing, text classification, machine learning, patient experience, patient-authored reviews, healthcare quality, patient care, psychology

## Abstract

**Background:**

Patients increasingly rely on web-based physician reviews to choose a physician and share their experiences. However, the unstructured text of these written reviews presents a challenge for researchers seeking to make inferences about patients’ judgments. Methods previously used to identify patient judgments within reviews, such as hand-coding and dictionary-based approaches, have posed limitations to sample size and classification accuracy. Advanced natural language processing methods can help overcome these limitations and promote further analysis of physician reviews on these popular platforms.

**Objective:**

This study aims to train, test, and validate an advanced natural language processing algorithm for classifying the presence and valence of 2 dimensions of patient judgments in web-based physician reviews: interpersonal manner and technical competence.

**Methods:**

We sampled 345,053 reviews for 167,150 physicians across the United States from Healthgrades.com, a commercial web-based physician rating and review website. We hand-coded 2000 written reviews and used those reviews to train and test a transformer classification algorithm called the Robustly Optimized BERT (Bidirectional Encoder Representations from Transformers) Pretraining Approach (RoBERTa). The 2 fine-tuned models coded the reviews for the presence and positive or negative valence of patients’ interpersonal manner or technical competence judgments of their physicians. We evaluated the performance of the 2 models against 200 hand-coded reviews and validated the models using the full sample of 345,053 RoBERTa-coded reviews.

**Results:**

The interpersonal manner model was 90% accurate with precision of 0.89, recall of 0.90, and weighted *F*_1_-score of 0.89. The technical competence model was 90% accurate with precision of 0.91, recall of 0.90, and weighted *F*_1_-score of 0.90. Positive-valence judgments were associated with higher review star ratings whereas negative-valence judgments were associated with lower star ratings. Analysis of the data by review rating and physician gender corresponded with findings in prior literature.

**Conclusions:**

Our 2 classification models coded interpersonal manner and technical competence judgments with high precision, recall, and accuracy. These models were validated using review star ratings and results from previous research. RoBERTa can accurately classify unstructured, web-based review text at scale. Future work could explore the use of this algorithm with other textual data, such as social media posts and electronic health records.

## Introduction

Patients increasingly turn to commercial physician rating and review websites to discuss their patient experiences and provide feedback to hospitals and providers [1,2]. Patient-authored reviews on these websites may capture factors of the patient experience not otherwise found in traditional patient experience surveys (eg, Press Ganey) or academic research (eg, interviews and questionnaires), such as insurance processing and appointment scheduling [3,4]. These websites have therefore gained increased attention among researchers seeking to better understand what patients care about and how commercial review data compare to other health care quality measures. For example, researchers analyzing commercial hospital reviews identified topics discussed by patients that were not covered in the current Hospital Consumer Assessment of Healthcare Providers and Systems survey, like nurse quality, staff compassion, and the technical aspects of care [3]. Other researchers found negative commercial reviews of surgeons focused on surgeon-independent factors, such as wait times and office staff, suggesting patients may consider factors beyond the patient-physician interaction when assessing quality [5].

Physician review websites potentially impact both patient choice and physician care quality. Some prospective patients rely on web-based physician ratings and reviews to help them choose physicians [4,6,7]. Research shows people prefer words to numbers and can easily comprehend review narratives over quantitative ratings [8]. Additionally, physicians use patient feedback conveyed in web-based written reviews to implement and improve quality measures, particularly related to patient communication [8].

The unstructured narrative text, however, presents a challenge for researchers seeking to make inferences from physician reviews. Methods previously used to identify patient judgments within written reviews include hand-coding [1] and dictionary-based approaches [9,10]. Hand-coding approaches, however, are time- and resource-intensive, which limits sample size [11]. Likewise, dictionary-based methods, such as Linguistic Inquiry and Word Count, use a context-independent bag-of-words approach, which may overlook misspellings, colloquialisms, and keywords and phrases not captured in prebuilt dictionaries [12,13].

In this paper, we present measures of precision, recall, and accuracy for an advanced natural language processing (NLP) algorithm, fine-tuned to identify the presence and valence of 2 dimensions of patient judgments in web-based physician reviews: interpersonal manner and technical competence. We use an algorithm called the Robustly Optimized BERT (Bidirectional Encoder Representations from Transformers) Pretraining Approach (RoBERTa), which we trained to classify our 2 judgment dimensions in written reviews and which has been successfully applied in other classification contexts (eg, Twitter) [13-15]. RoBERTa’s novelty is in its transformer-based, bidirectional, context-aware approach, wherein it is pretrained on a large corpus of text but is fine-tunable for many NLP tasks, including text classification [15,16]. We validate this algorithm by correlating results with review star ratings and by comparing results with those found in prior literature.

## Methods

### Data Collection

**Figure 1:**
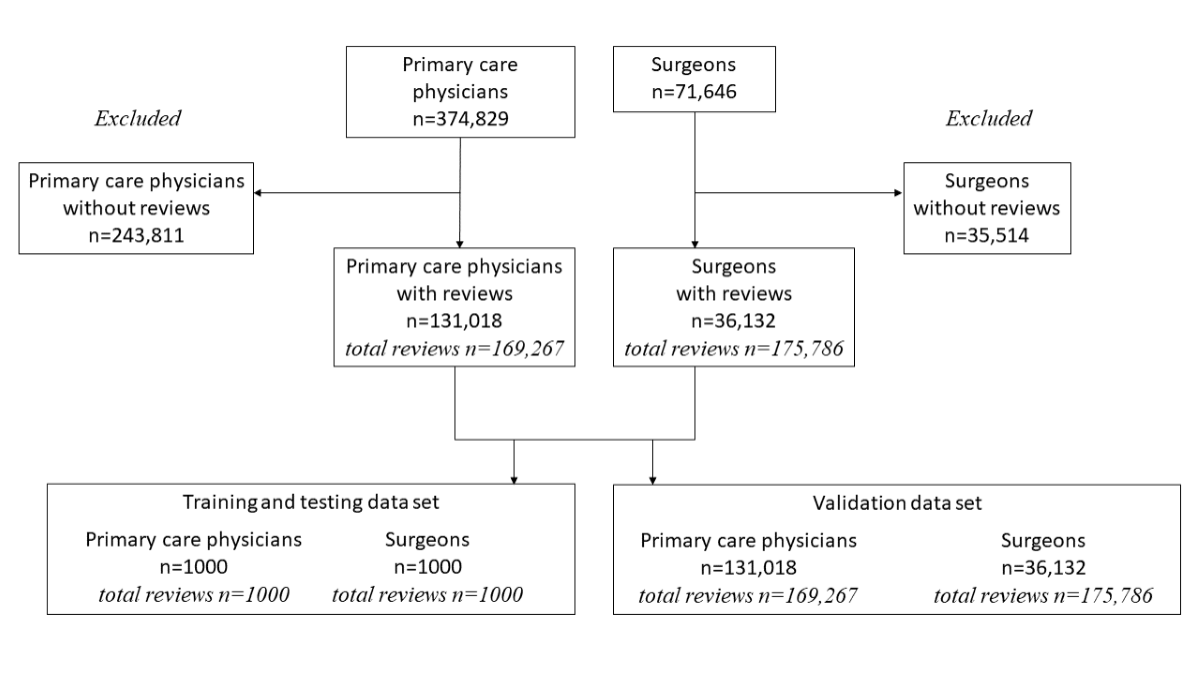
Sample selection flow chart.

We scraped physician profiles, ratings, and review data published on Healthgrades.com in April 2020. We collected primary care physician profiles associated with family medicine, internal medicine, and pediatrics, and surgeon profiles associated with general surgery; orthopedic surgery; and cosmetic, plastic, and reconstructive surgery. Healthgrades.com has a physician profile for every US physician with an active profile listed on the National Provider Identifier Registry [17]. In addition to physician profile characteristics, we scraped rating information and up to 20 of the most recent written reviews per physician. On Healthgrades.com, patients can elect to submit a star rating alone (ie, 1-5 stars, no fractions) or a star rating accompanied by a written review. The study was approved by the Duke University institutional review board and all data collected were publicly available and aggregated for research purposes. Our final sample included 345,053 reviews submitted for 167,150 physicians (primary care physicians and surgeons). Figure 1 shows a flow chart of our sample selection.

### Coding Reviews for Interpersonal Manner and Technical Competence

#### Hand-Coding the Training Data

We first cleaned the text to convert non–ASCII-encoded characters to ASCII characters; examples included some apostrophes, dashes, and letters with accents (eg, blasé). We then trained our classification algorithm using a gold standard data set of rigorously hand-coded physician reviews [11]. We purposely sampled 2000 random reviews for equal representation of primary care physicians and surgeons, female and male physicians, and low-star (≤3 stars) and high-star (≥4 stars) review ratings. We achieved high interrater reliability with a subset of 300 double-coded reviews (Cohen κ range 0.74-0.85), before proceeding to independently code the remaining reviews.

**Figure 2:**
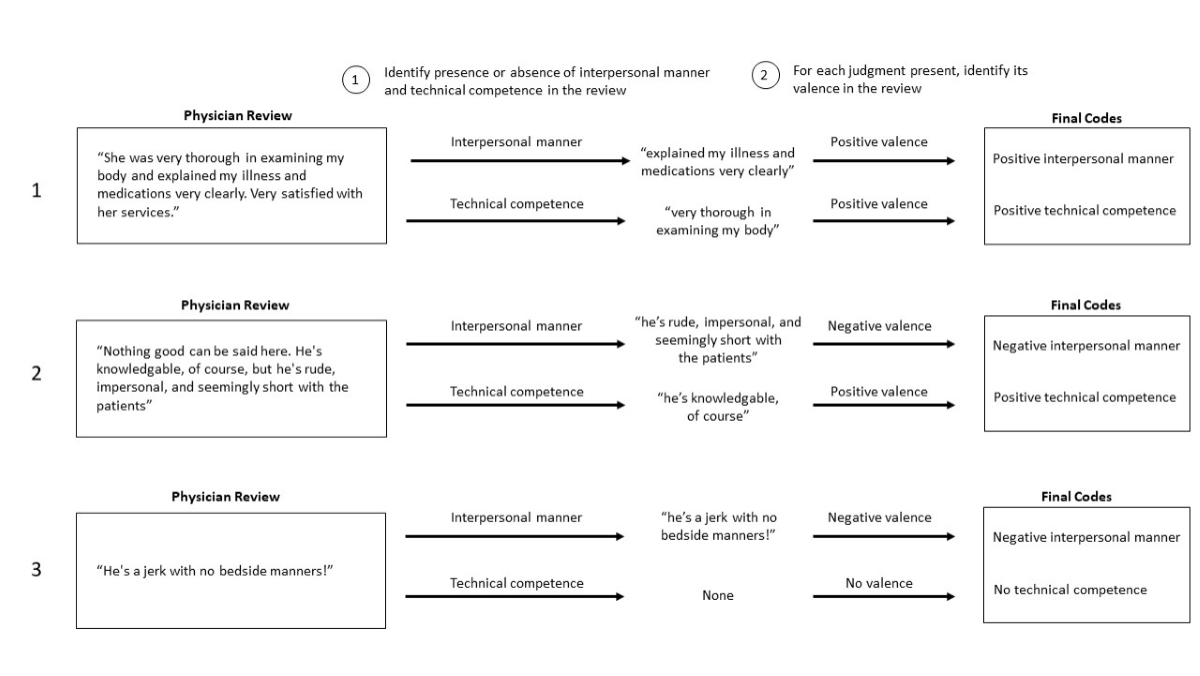
Diagram showing how real physician reviews were hand-coded for the presence and valence of interpersonal manner and technical competence.

We coded each review for the presence or absence of interpersonal manner and technical competence. Reviews could be coded for the presence of only 1 dimension, both dimensions, and neither dimension. Once we indicated the presence of a judgment dimension, we coded the valence of the judgment as positive or negative. If we did not code the presence of a judgment dimension, we would not have a valence indicated for that judgment. Figure 2 provides a diagram with illustrative examples showing how we hand-coded the presence and valence of the 2 judgment dimensions.

#### Training and Testing the Algorithm

To code patients’ interpersonal manner and technical competence judgments in the sample of 345,053 reviews, we first used our hand-coded data to train RoBERTa, a transformer classification model. Transformers are neural network systems that use vectors to capture the meanings of words in context and are the main architecture underlying advanced NLP models [13,18]. These state-of-the-art NLP models improve upon prior NLP classification approaches, such as those based on dictionaries or fixed embeddings [13]. Specifically, RoBERTa builds bidirectional context-aware embeddings such that the vector representing the word changes depending on its context in the text [13,19]. RoBERTa is pretrained on Book Corpus (800 million words) and English Wikipedia (2500 million words), and can be fine-tuned with one additional level of training data for specific classification tasks [20]. We implemented RoBERTa with assistance from simple transformers [21], a wrapper library for the HuggingFace Transformers library [22]. In our study, each review used to train RoBERTa has its own sequence embedding and helps fine-tune the model to code the reviews for the presence and valence of interpersonal manner and technical competence.

We fine-tuned 2 multiclass classification models, 1 for classifying interpersonal manner and 1 for technical competence. We tuned each model using 1600 (80%) reviews randomly sampled from our hand-coded training data. We completed 6 iterations through our training data for both models. We used a test data set, or a set of 200 (10%) hand-coded reviews held out of the training data, to evaluate each model’s fit on the training data set while further fine-tuning the model. Finally, we used a new set of 200 (10%) hand-coded reviews to provide an unbiased evaluation of the classification performance of each fully trained model for patients’ interpersonal manner and technical competence judgments. Training and evaluation batch sizes for both models were 1024 sequences; both models used 6 training epochs, with final epoch running losses <0.01.

After training and testing the 2 models using the 2000 hand-coded reviews, we applied the fully trained models to all the reviews in our data set, including the 2000 reviews we hand-coded. This resulted in a data set with 345,053 reviews coded by RoBERTa for the presence and valence of interpersonal manner and technical competence judgments. We used Python and Google Colab to train RoBERTa on our judgment classification tasks and code our full sample of reviews.

## Results

### Evaluating the Accuracy of the 2 Classification Models

Our 2 classification models were highly accurate. The out-of-sample predictive accuracy for the interpersonal manner model was 90% with a weighted *F*_1_-score of 0.89 (range 0.82-0.95), precision of 0.89 (range 0.85-0.94), and recall of 0.90 (range 0.80-0.96). The out-of-sample predictive accuracy for the technical competence model was also 90%, with a weighted *F*_1_-score of 0.90 (range 0.90-0.92), precision of 0.91 (range 0.88-0.95), and recall of 0.90 (range 0.85-0.95). Table 1 details the classification performance metrics for the interpersonal manner and technical competence models.

**Table 1:** Fine-tuned transformer classification performance for interpersonal manner and technical competence judgments.

Classification model and valence^a^			Precision^b^		Recall^c^		*F*_1_-score^d^
**Interpersonal manner model**							
	No interpersonal manner	0.85		0.80		0.82	
	Negative interpersonal manner	0.88		0.89		0.88	
	Positive interpersonal manner	0.94		0.96		0.95	
	Accuracy	—^e^		—		0.90	
	Macro avg	0.89		0.88		0.88	
	Weighted average	0.89		0.90		0.89	
**Technical competence model**							
	No technical competence	0.88		0.91		0.90	
	Negative technical competence	0.95		0.85		0.90	
	Positive technical competence	0.89		0.95		0.92	
	Accuracy	—		—		0.90	
	Macro average	0.91		0.90		0.90	
	Weighted average	0.91		0.90		0.90	

^a^Classification performance is based on a comparison to an evaluation data set of 200 reviews hand-coded by our team of researchers.

^b^Precision: number of true positives divided by the sum of true positives and false positives.

^c^Recall: number of true positives divided by the sum of true positives and false negatives.

^d^*F*_1_-score: harmonic mean of precision and recall, given by 
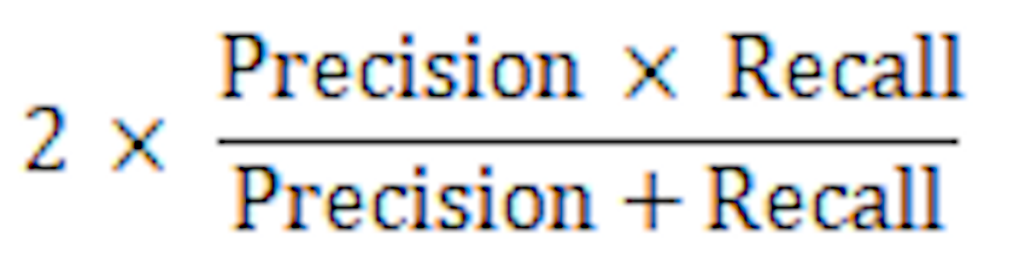
 [14].

^e^Not applicable.

### Comparing Reviews Coded by RoBERTa and by Hand

As part of our final sample of 345,053 reviews, the 2000 hand-coded reviews from our training data set were recoded by RoBERTa. The interrater reliability between our hand-coding and RoBERTa was Cohen κ =0.96 for both interpersonal manner and technical competence. Comparing the RoBERTa codes with the original hand codes for these reviews, we found only 107 (5.4%) reviews had coding discrepancies. Of those, 49 (2.5%) reviews had the same interpersonal manner code but different technical competence code, 57 (2.9%) reviews had the same technical competence code but different interpersonal manner code, and 1 (0.05%) review had both different interpersonal manner and technical competence codes. Table 2 shows illustrative examples of coding discrepancies in our RoBERTa-coded reviews and our hand-coded reviews from the training data set.

**Table 2:** Illustrative examples of discrepancies in reviews coded by the RoBERTa^a^ and by hand and reasoning underlying the discrepancies.

Review	RoBERTa coding		Hand-coding with reasoning	
	Interpersonal manner	Technical competence	Interpersonal manner	Technical competence
#metoo. He knows what he did.	0^b^	0	–^c^ (Hints at sexual assault by the physician)	0
When someone is scared, I Think the Dr. should try to comfort them instead of telling them that when I finish this procedure, you will no longer be my patient . I’ll refer you to someone else. So I wish him the best.	+^d^	0	– (Feels physician did not provide comfort)	0
very rude receptionists answering phones, unhelpful, and sarcastic, they should be replaced ! they cant be bothered especially trish! very rude!	–	0	0 (Discusses the interpersonal manner of the staff, not the physician)	0
Walking on the second day. My daughter also! I would send anyone I know his way... We’re from out of town and after using a local doctor here and having to do it all over again the experience was amazing and recovery was shorter	0	0	0	+ (Perceives treatment as a success)
Awful experience! Thanks to another doctor that happened to see the urgency of my condition, I got the help that I needed. Had it been left to Dr. C^e^, God only knows where I’d be today. Avoid him!!!	0	+	0	– (Perceives poor physician decision-making)
Excellent !!!!!! You will never ever find a better M.D. Caring, so professional an excellent surgeon with compassion??	+	+	+	0 (Does not discuss physician’s expertise, treatment, or outcomes)
Very similar to all the one star ratings, if 0 stars were an option I’d choose that. The follow up on patients are non-existent, which makes it very obvious that the surgeon just wants $. The staff is always rude. I wish they would treat their patients and their family members how they would like their own to be treated. My mom has had two infections where they removed her lymph nodes after trying to call them about this several times, we took her to a different doctor to have the site drained	0	0	– (Feels physician prioritized money over care)	– (Critiques physician’s lack of follow-up care)

^a^RoBERTa: Robustly Optimized BERT (Bidirectional Encoder Representations from Transformers) Pretraining Approach.

^b^“0” indicates no judgment coded.

^c^“–” indicates negative judgment coded.

^d^“+” indicates positive judgment coded.

^e^Physician surname reproduced here by only its first letter.

### Testing the Validity of the 2 Classification Models

#### Overview

We tested the validity of our classification models using the full sample of RoBERTa-coded reviews. We validated our models in 2 ways. First, we related our valence coding with the review star ratings (ie, 1- to 5-star ratings submitted with each review), with the expectation that positive-valence judgments would be associated with higher star ratings and negative-valence judgments would be associated with lower star ratings. Second, we compared our findings with prior literature on patients’ judgments of physicians in web-based physician reviews.

#### Testing for Associations Between Judgment Valence and Star Ratings

**Figure 3:**
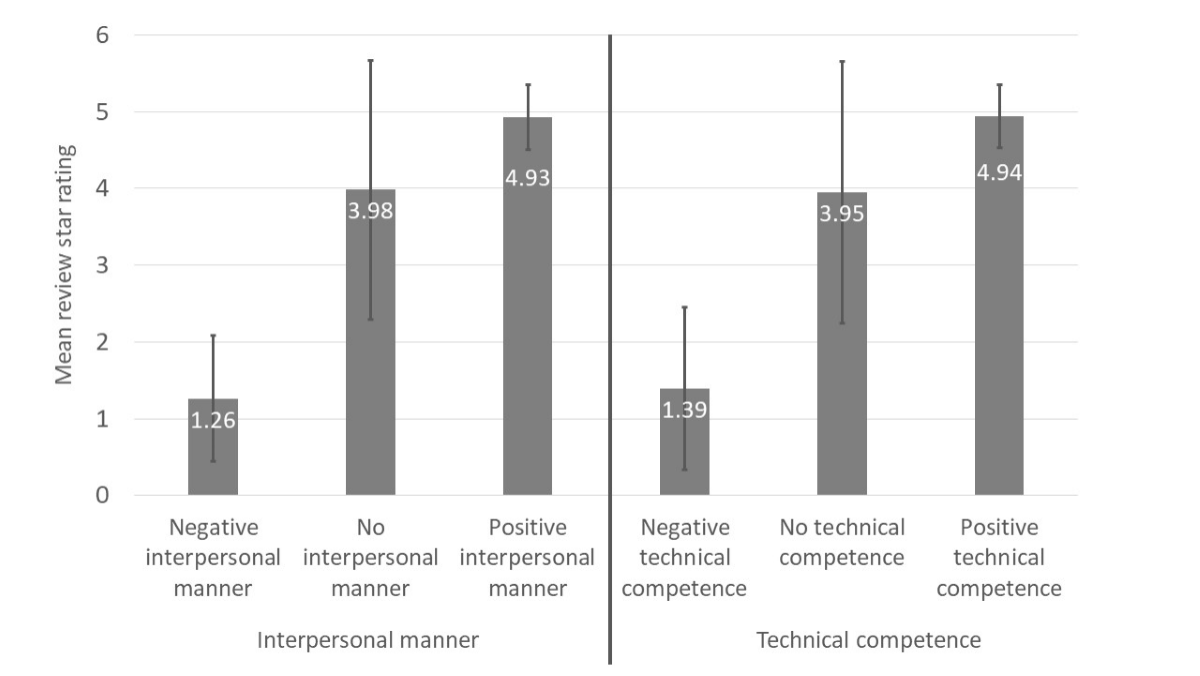
Mean review star ratings with SDs, for reviews with negative, no, and positive interpersonal manner or technical competence.

Using multilevel linear regressions, we analyzed associations between patients’ interpersonal manner and technical competence judgment valences and review star ratings. We found evidence of construct validity: positive valences for both interpersonal manner and technical competence were significantly positively associated with review star ratings whereas negative valences for both judgment dimensions were significantly negatively associated with review star ratings. Compared with reviews with no or negative judgment, reviews with positive interpersonal manner were associated with 1.82 (95% CI 1.81-1.83; *P*<.001) more stars, and reviews with positive technical competence were associated with 1.50 (95% CI 1.49-1.51; *P*<.001) more stars. In contrast, compared with reviews with no or positive judgment, reviews with negative interpersonal manner were associated with 3.30 (95% CI –3.31 to –3.29; *P*<.001) fewer stars and reviews with negative technical competence were associated with 3.00 (95% CI –3.01 to –2.98; *P*<.001) fewer stars. Figure 3 displays mean review star ratings for each judgment dimension.

#### Testing Whether the Models Reproduce Prior Findings

One study analyzing 712 reviews determined that 69% of interpersonal manner reviews and 80% of technical competence reviews were positive [1]. We identified a similar pattern of majority positive reviews; 207,327 (81%) reviews mentioning interpersonal manner and 178,705 (82%) reviews mentioning technical competence were positive. Another study reported physicians who received reviews with interpersonal manner language were at least 2.39 times more likely to receive a 5-star review rating [9]. We similarly found physicians who received interpersonal manner reviews had 1.69 times the odds of receiving a 5-star review rating (95% CI 1.65-1.73; *P*<.001). When controlling for physician gender, specialty, age, and practicing state, as well as review word count, physicians with interpersonal manner reviews continued to have higher odds of receiving a 5-star review rating (odds ratio [OR] 2.22, 95% CI 2.17-2.28; *P*<.001).

Prior research also showed female physicians, compared with male physicians, had higher odds of receiving reviews mentioning interpersonal manner [9,10]. Our findings supported these results: we determined female physicians had 1.56 times the odds of receiving a review mentioning interpersonal manner than male physicians (95% CI 1.53-1.59; *P*<.001). When controlling for physician specialty, age, practicing state, and review word count, female physicians still had significantly higher odds of receiving an interpersonal manner review (OR 1.19, 95% CI 1.17-1.22; *P*<.001).

One group of investigators demonstrated female physicians were more likely than male physicians to receive both reviews praising and reviews criticizing their interpersonal manner [10]. Consistent with these results, we found female physicians had 1.40 times the odds of receiving a negative review about their interpersonal manner than male physicians (95% CI 1.36-1.44; *P*<.001). This significant difference remained when including controls (OR 1.25, 95% CI 1.21-1.29; *P*<.001). Female physicians also had 1.18 times the odds of receiving a positive interpersonal manner review than male physicians (95% CI 1.15-1.20; *P*<.001); however, this gender difference was not significant when including controls (OR 1.02, 95% CI 1.00-1.04; *P*=.05).

Likewise, another study concluded that highly rated male physicians were 1.48 times more likely to receive reviews describing technical competence whereas highly rated female physicians were 2.11 times more likely to receive reviews describing interpersonal manner [23]. We found similar results: Highly rated female physicians had 1.76 higher odds of receiving an interpersonal manner review (95% CI 1.72-1.81; *P*<.001), which was still significant after including controls (OR 1.25, 95% CI 1.22-1.29; *P*<.001). Highly rated male physicians had 1.33 higher odds of receiving a technical competence review (95% CI 1.30-1.36; *P*<.001); however, with controls, this gender difference flipped, such that highly rated females were more likely to receive a technical competence review (OR 0.95, 95% CI 0.93-0.98; *P*<.001).

## Discussion

### Principal Results

Our 2 classification models identified the presence and valence of patients’ interpersonal manner and technical competence judgments with high precision, recall, and accuracy. Our models identified these 2 judgment dimensions from a broad training data set inclusive of reviews for female and male physicians, for primary care physicians and surgeons, and for low- and high-star-rated reviews.

Our interpersonal manner and technical competence models underperformed in classifying reviews with no judgment and negative valence relative to reviews with positive valence. However, the overall predictive accuracy of both models (90%) was higher than the rate of hand-coding agreement among the 4 investigators (Cohen κ=0.84 and 0.77 for interpersonal manner and technical competence, respectively).

Our models produced classification metrics comparable to those found in other studies that use fine-tuned RoBERTa algorithms for coding tasks. For example, researchers who used RoBERTa to detect polarizing versus nonpolarizing rhetoric in tweets written by Congress members reported a model with 90% predictive accuracy and a weighted *F*_1_-score of 0.90 [13]. They compared their results to the Valence Aware Dictionary and Sentiment Reasoner, a dictionary-based sentiment analysis model, which demonstrated a 68% accuracy and *F*_1_-score of 0.75. Another study that used RoBERTa to classify 5 classes of mental illness in Reddit posts reported a model with an *F*_1_-score of 0.86 [24]. These researchers showed RoBERTa outperformed both BERT and long short-term memory, a nontransformer neural network text classifier (86% accuracy vs 82% and 72%). Last, researchers who forecasted star ratings from physician reviews written on RateMDs.com demonstrated an 84.6% accuracy and a mean *F*_1_-score of 0.83 with their RoBERTa model, which outperformed other NLP models [25]. In comparison, our interpersonal manner and technical competence models were each 90% accurate with weighted *F*_1_-scores of 0.89 and 0.90, respectively.

Although we did not compare our own RoBERTa models to other NLP algorithms, our accuracy scores perform equal to or better than prior methods used to code patients’ judgments in web-based physician reviews. For example, in one study, investigators who hand-coded reviews for 4 broad thematic categories, including interpersonal manner and technical competence, reported an interrater reliability range of κ=0.8-1.0 [1]. Another study, which used dictionary-based text analysis to code for positive and negative soft skills reported a mean accuracy of 0.76 (range 0.42-0.92) [10]. The rates of hand-coding agreement for interpersonal manner and technical competence among our own 4 investigators were Cohen κ=0.84 and 0.77, respectively. Last, research has shown RoBERTa outperforms both other pretrained models and traditional machine-learning models (eg, support vector machines and random forests) when used for text classification tasks in the health domain [16,26].

Our NLP classification models overcome several limitations of prior research using hand-coding and dictionary-based methods to identify the prevalence and valence of patient judgments in web-based physician reviews. Hand-coding, although considered the gold standard, is time-intensive, which limits scalability. Prior studies using multiple coders could only analyze data from sample sizes of fewer than 1000 physician reviews [1,27]. Dictionary-based approaches enable analysis of larger samples but are restricted to the keywords contained in their dictionaries. Dictionary-based or bag-of-words models may overlook misspellings and jargon (eg, “he butchered my surgery”), leading to false negatives. They may also misidentify judgments about nonphysician staff (eg, front desk worker and nurse) or pick up words that have different meanings in different contexts (eg, “he is thorough in his examinations” vs “she gives thorough explanations”), leading to false positives. Dictionary-based models also have difficulty distinguishing between words used positively or with negations (eg, “she was smart” vs “she was not smart”), which complicates valence estimates. Prior research on physician reviews using dictionary-based models could not determine valence and only coded reviews that contained at least 1 preselected dictionary keyword [9,10,28,29].

We validated our classification models by examining associations between our coded judgment valence and review star ratings and by comparing our coded judgments to findings from prior studies. We found positive interpersonal manner and technical competence judgments were associated with higher review star ratings whereas negative judgments were associated with lower review star ratings. Additionally, we found similar patterns of results with other studies that have examined the presence and valence of patients’ judgments in web-based physician reviews. Future research should examine how both interpersonal manner and technical competence judgments vary depending on both physician gender and specialty.

We demonstrate that fine-tuning RoBERTa classification models to code patients’ interpersonal manner and technical competence judgments in web-based physician reviews offers a scalable, reliable, and accurate method for analyzing unstructured textual review data. To our knowledge, we are the first to use an advanced NLP algorithm to code a large data set of web-based physician reviews for patients’ judgments. This algorithm successfully coded web-based physician reviews, suggesting that RoBERTa may also be used to code similar unstructured text, including reviews from other commercial physician review websites (eg, RateMDs and ZocDoc) and from traditional Press Ganey patient-experience surveys. Whereas research has begun to use BERT-based models to extract health care insights from triage notes and medical records [30-32], future research is needed to ascertain the effectiveness of RoBERTa models with more far-afield text, such as crowdfunding campaigns, social media posts, and recommendation letters.

We acknowledge patients’ judgments of their physicians’ technical competence should be taken with caution. Prior research has shown weak correlations between patients’ assessments of technical care quality and evidence-based indicators from clinical records [33]. Certifying boards and professional societies are better equipped to assess physicians’ technical skills, such as knowledge of diagnostic and therapeutic advances [34]; however, patients’ written reviews may be useful in offering reports of what actually occurred during clinical encounters, such as whether the physician checked their blood pressure or offered a flu vaccine [35]. Thus, for patient reviews to improve clinical care quality, future classification models for technical competence may consider focusing more narrowly on patient reports of technical processes rather than general perceptions of physicians’ technical skills.

### Limitations

Our study has 3 broad limitations, which arise from both the training data and the algorithm output. First, despite representing a small sample of our data set, our 2000 hand-coded reviews took substantial time and resources. It is possible that future studies can use smaller samples to train RoBERTa for classification tasks while maintaining high accuracy. Although more research is needed, we explored this idea by re-fine-tuning our models with gradually smaller training data sets (ie, n=1000, 500, and 250 reviews). For both interpersonal manner and technical competence, accuracy decreased with smaller training samples (interpersonal manner: 90%, 87%, 82%, and 64%; technical competence: 90%, 86%, 72%, and 64%). This brief example shows that future researchers could use 1000, possibly 500, but likely not 250 hand-coded reviews to train RoBERTa for multiclass classification. However, researchers should consider the implications of not only accuracy but also precision and recall for each code.

Second, our RoBERTa models were only as good as our training data, and our training data was imperfect. Although hand-coding is considered the gold standard and team members rigorously followed a coding framework, biases in how individual coders identified patients’ interpersonal manner and technical competence judgments may have influenced the RoBERTa models. The imperfect interrater reliability present within the hand-coded data set is evidence of differences between coders, which may have complicated the fine-tuning of the models. Additionally, the 2000 hand-coded reviews represented only 0.6% of all reviews in the final data set; thus, our models could have been overfitted to this relatively small training data set.

We also excluded the classification of certain reviews in our training data. Because of our own language barriers, we trained the models on reviews only written in English. Reviews written in other languages, such as Spanish, were not translated and thus received codes of no interpersonal manner and no technical competence, despite potentially describing either judgment dimension. Reviews written in languages other than English, however, represented a small proportion of the total reviews in our sample. In addition, we only trained the models to classify patient judgments of physicians’ interpersonal manner and technical competence, ignoring other judgments. Other judgments categorized as neither interpersonal manner nor technical competence included global remarks (eg, “would definitely recommend to others!” or “the worst”) and system-level comments about the office, staff, or other aspects of the health care experience (eg, “dingy building” or “his assistant was the most wonderful person I have ever met”).

Third, our RoBERTa models had limitations. Although RoBERTa offers a more advanced NLP algorithm than dictionary-based methods, the algorithm may still not recognize cultural jargon. The first illustrative example of Table 2, in which RoBERTa did not recognize the connotation of the #metoo reference, demonstrates this limitation. Moreover, because the RoBERTa algorithm was not trained on a prebuilt dictionary but on a reference set of hand-coded reviews, it is difficult to determine specific words and phrases the models used when classifying interpersonal manner and technical competence judgments. This transparency limitation, often called “black box AI” is a common problem with deep learning algorithms that create their own neural networks for categorization [36,37].

Despite these limitations, practical benefits of applying advanced NLP algorithms, like RoBERTa, to physician reviews include the enhanced capability to review feedback on what patients like and dislike about their medical encounters at clinician, department, or hospital levels; assistance in discerning differences in physician reviews received in traditional versus web-based surveys; and support in identifying patient biases, if any, corresponding with physician demographics. The benefits of these large language models also extend beyond insights from physician reviews. For example, advanced NLP models can improve patient care through medical information retrieval from medical literature, drug databases, and treatment guidelines; and through personalized clinical decision support by analyzing relevant patient data, such as medical histories, test results, and clinician notes. These models can also reduce physician workload via documentation assistance.

### Conclusion

We coded a large data set of web-based physician reviews for the presence and valence of patients’ interpersonal manner and technical competence judgments using RoBERTa, a pretrained NLP classification algorithm. We trained and tested our models using a gold standard data set of hand-coded reviews and demonstrated that our models accurately and reliably coded interpersonal manner and technical competence. We also validated the algorithm by comparing our RoBERTa-coded data set with review star ratings and results from prior literature. The RoBERTa algorithm overcomes text analysis limitations present in previous work by identifying patient judgments in a broad range of physician reviews accurately and at scale. Potential benefits of advanced NLP models pertain to web-based physician reviews and beyond, from helping physicians more efficiently assess patient feedback to improving physicians’ workload and patient care.

## Abbreviations

BERT: Bidirectional Encoder Representations from Transformers
NLP: natural language processing
OR: odds ratio
RoBERTa: Robustly Optimized BERT Pretraining Approach
